# Effect of CeO_2_-Reinforcement on Pb Absorption by Coconut Coir-Derived Magnetic Biochar

**DOI:** 10.3390/ijms24031974

**Published:** 2023-01-19

**Authors:** Yujia Yang, Rui Shan, Yaoxin Xiao, Fengxiao Zhao, Haoran Yuan, Yong Chen

**Affiliations:** 1 Guangzhou Institute of Energy Conversion, Chinese Academy of Sciences (CAS), Guangzhou 510640, China; 2 CAS Key Laboratory of Renewable Energy, Guangdong Provincial Key Laboratory of New and Renewable Energy Research and Development, Guangzhou 510640, China

**Keywords:** CeO_2_-magnetic biochar, magnetic adsorbent, Pb^2+^ removal, absorption mechanism

## Abstract

Magnetic separable biochar holds great promise for the treatment of Pb^2+^-contaminated wastewater. However, the absorption effect of unmodified magnetic biochar is poor. Considering this gap in knowledge, CeO_2_-doped magnetic coconut coir biochar (Ce-MCB) and magnetic coconut coir biochar (MCB) for Pb^2+^ absorption were prepared by the impregnation method, and the efficiency of Ce-MCB for Pb^2+^ absorption was evaluated in comparison with MCB. Conducting the absorption experiments, the study provided theoretical support for the exploration of the absorption mechanism. The quantitative analysis exposed that the enhanced absorption capacity of Ce-MCB was attributed to the increase in oxygen-containing functional groups and mineral precipitation. The Langmuir and Freundlich isotherm model showed that Ce-MCB is a suitable adsorbent for Pb^2+^. The absorption characteristics of Ce-MCB was fit well with the pseudo-second-order (PSO) and Langmuir models, which revealed that the absorption of Pb^2+^ in water was monolayer chemisorption with a maximum theoretical adsorption capacity of 140.83 mg·g^−1^. The adsorption capacity of Ce-MCB for Pb(II) was sustained above 70% after four cycles. In addition, the saturation magnetization intensity of Ce-MCB was 7.15 emu·g^−1^, which was sufficient to separate out from the solution. Overall, Ce-MCB has wide application prospects in terms of biomass resources recycling and environmental conservation.

## 1. Introduction

The widespread occurrence of pollution in surface water and groundwater, especially toxic elements, due to human activities may be exceptionally detrimental to the ecosystem [1]. Heavy metals are persistent in the environment and accumulate in organisms through the food chain, negatively impacting the growth of plants and animals. Pb^2+^ is one of the toxic and harmful pollutants present in industrial wastewater [2]. Over the last two centuries, Pb^2+^ has been widely used in production and daily life such as those from battery production, electroplating, mining, metal smelting, and the use of irrigation, which has proliferated the spread of lead into the environment [3]. Pb can enter the human body mainly through drinking water and food, which creates severe toxicity to human organs. High concentrations of Pb cause adverse effects such as anemia, kidney dysfunction, and gastric cancer [4,5]. The Agency for Toxic Substances and Disease Registry (IARC) identified Pb^2+^ as one of the most hazardous elements [6]. 

Currently, prevailing technologies have been adopted for controlling Pb^2+^ pollution, including electrochemical treatment technologies [7], flocculation [8], coagulation [9], absorption [10], and precipitation [11]. Absorption is regarded as one of the most promising methods, which is cost-effective, technically effective, and environmentally friendly. Several adsorbents such as microplastic particles [12], hydrogel balls [13], mineral [14], slag [15], and nano-adsorbent [16] have been used to treat polluted environments. However, disadvantages such as high preparation cost, difficult separation, and low efficiency limit their application.

Biochar as a carbon-rich material is considered a cost-effective and environmentally friendly adsorbent, and has received more concern because of its simple preparation, wide range of sources, low price, and strong effect. It has sufficient selectivity and applicability in removing toxic heavy metal ions from various water environments. [17]. Coconut coir is a typical abundant and inexpensive agricultural waste, which is still mainly used for the production of low value-added products, with only a small amount for biochar production. More than 420,000 tons coconuts are produced annually in China, and the production of coconut skin can reach 250 million pieces [18]. In the current study, Li et al. [19] reported that coconut-coir-derived biochar can provide binding sites for Pb^2+^. However, the ability of pristine biochar to remove heavy metals is not impressive, and it is difficult to separate from aqueous solution. In general, biochar magnetically treated by means of Fe^2+^/Fe^3+^ solution or natural hematite can be easily separated from the solution by external magnets, but magnetic biochar without modification has poor adsorption performance [20]. As magnetic biochar has a low adsorption capacity, it was modified to expand the absorption capacity of the biochar while retaining the magnetic properties. 

There are few studies that have reported the use of cerium-nitrate-modified magnetic biochar for Pb^2+^ removal in aqueous environments. Previous research has shown that the absorbents have been modified by loading transition metal oxides and alkaline earth mental oxides on the surface of biochar [21,22]. CeO_2_ is one of the most industrially useful rare earth metal oxides with low toxicity, and its pure metal has a high emission threshold to the environment, which makes the absorbent less toxic [23,24]. CeO_2_ nanoparticles, as a new absorption material, have great potential in heavy metal removal. In Malinee’s study, samaria-doped ceria nanopowder had a high removal capacity at low Pb^2+^ concentrations, but was confined by the aggregation of nanoparticles. In addition, the theoretical maximum absorption capacity of the adsorbent is 23 mg·g^−1^ [25]. Market-Borayo reported the efficiency of Pb^2+^ absorption by CeO_2_ nanoparticles, while the results were comparable in a previous study [15]. Recillas et al. [26] synthesized CeO_2_ nanocrystal precipitates, where they showed strong absorption capacities of Pb^2+^ up to 189 mg·g^−1^. Nevertheless, CeO_2_ nanocrystal precipitates presented a high phytotoxicity, which undoubtedly poses a great threat to aqueous organisms and human health. In order to fully utilize the excellent adsorption performance of CeO_2_, its high toxicity disadvantage needs to be overcome as a priority. In this study, we propose the stabilization of CeO_2_ on magnetic carbon materials by high-temperature calcination to make it easy to separate.

**Scheme 1:**
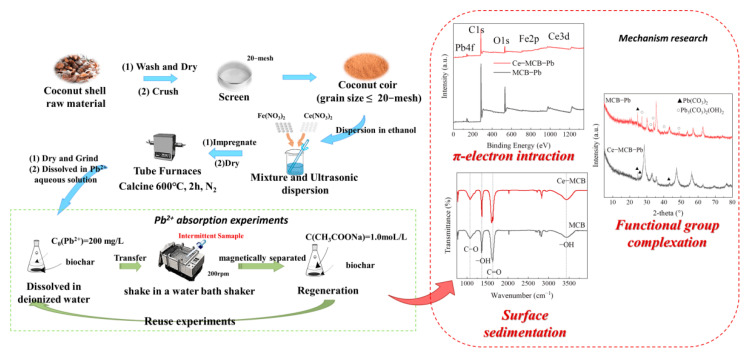
Schematic illustration of biochar preparation and adsorption experiments.

In the study, the synthesis of magnetic biochar doped with CeO_2_ (noted as Ce-MCB) via a simple impregnation method is reported to improve the adsorption performance of Pb^2+^. The physicochemical properties (surface morphology, pore structure, chemical composition, magnetic properties, etc.) of magnetic biochar (MCB) and CeO_2_-doped magnetic biochar (Ce-MCB) were explored. Moreover, the effects of time dependence, pH, and Pb^2+^ concentration on the absorption capacity of two magnetic biochars were also analyzed. The corresponding models of isotherms and kinetics were further developed to describe the adsorption process. In terms of the adsorption mechanism, XPS, XRD, and FTIR analyses were performed on the biochar before and after adsorption to qualitatively explore the adsorption mechanism, and the adsorption rate of each component was quantified by the acid washing method. The experimental procedure is shown in Scheme 1. 

## 2. Results and Discussion

### 2.1. Morphology Characteristics

In order to understand the surface characteristics and elemental composition of Ce-MCB, a systematic characterization of the as-prepared magnetic adsorbent was performed. Additionally, to investigate the effect of Ce addition on the adsorption performance, MCB was also prepared and tested for physicochemical properties.

**Figure 1:**
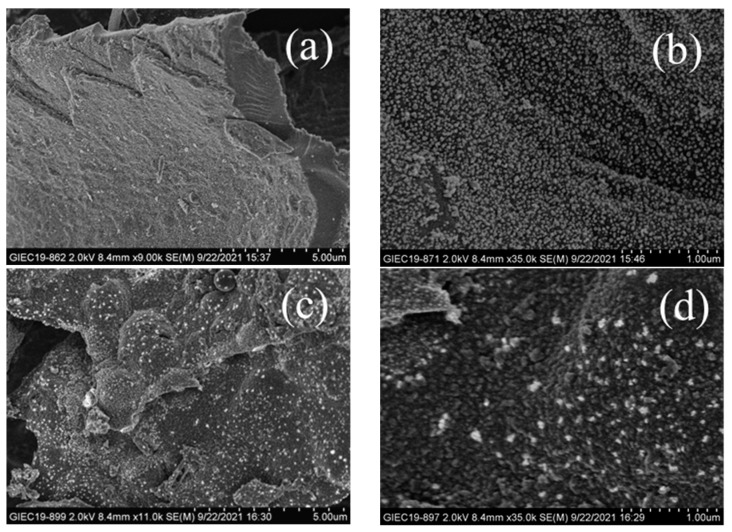

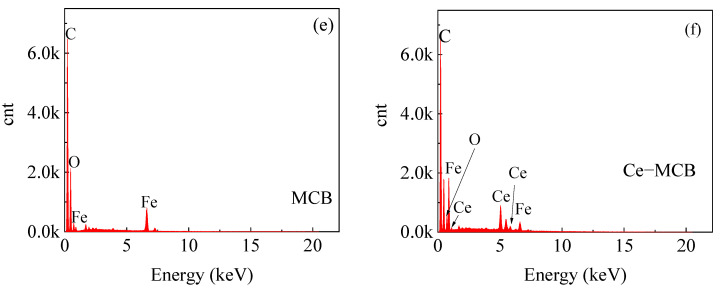
SEM image of MCB (**a**,**b**) and Ce-MCB (**c**,**d**), EDX spectra of MBC (**e**) and Ce-MCB (**f**).

The morphology and composition of MCB and Ce-MCB were carried out by SEM. As can be seen from Figure 1a–d, Ce-MCB had a richer pore structure compared to MCB. Meanwhile, Ce-MCB also exhibited a rougher surface, which affords a vast range of available surface-active sites. These differences suggest that the added Ce(NO_3_)_3_, which can act as a chemical activator during pyrolysis at high temperatures, lead to a significant change in the textural properties of the biochar [27]. In addition, the presence of Fe on MCB and the presence of Ce and Fe on Ce-MCB were detected by the SEM-EDX test, which indicates the successful preparation of the adsorbents (Figure 1e,f).

**Figure 2:**
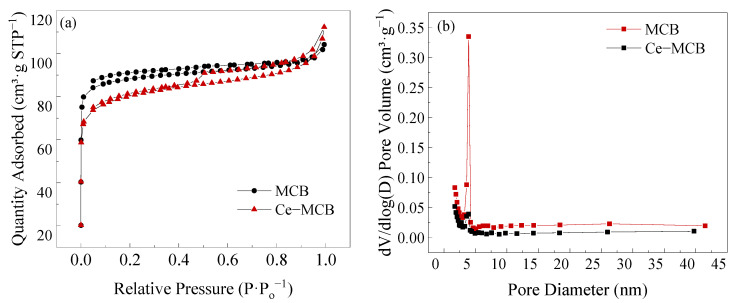
The N_2_ adsorption-desorption curves (**a**) and pore size distribution (**b**) of MCB and Ce-MCB.

The specific surface area (SSA) and pore size distribution of biochar were analyzed and the results are shown in Figure 2 and Table 1. As seen from Figure 2a, the N_2_ adsorption isotherms of both samples belonged to the type IV mode with a clear hysteresis loop according to the International Union of Pure and Applied Chemistry (IUPAC) classification, which indicates the presence of mesoporous structures in them [28,29,30]. The curve of MCB was open, which is attributed to the difficulty of completely desorbing the gas from pores of MCB after adsorption due to the complex of the pore structure for biochar and the tendency to produce elastic or ink-bottle-like pores [31,32]. The BET surface area of Ce-MCB was 246.32 m^2^·g^−1^, which was higher than that of MCB (234.35 m^2^·g^−1^). As shown in the BJH pore size distribution curve (Figure 2b), a large abundance of microporous and mesoporous structures were present in the prepared adsorbents. Notably, the quantity of Ce-MCB mesopores was significantly increased compared to MCB, especially in the size range of 2.5–5 nm, which suggests that the incorporation of cerium oxide had a certain activating effect on the development of pore structure in biochar [33,34].

**Figure 3:**
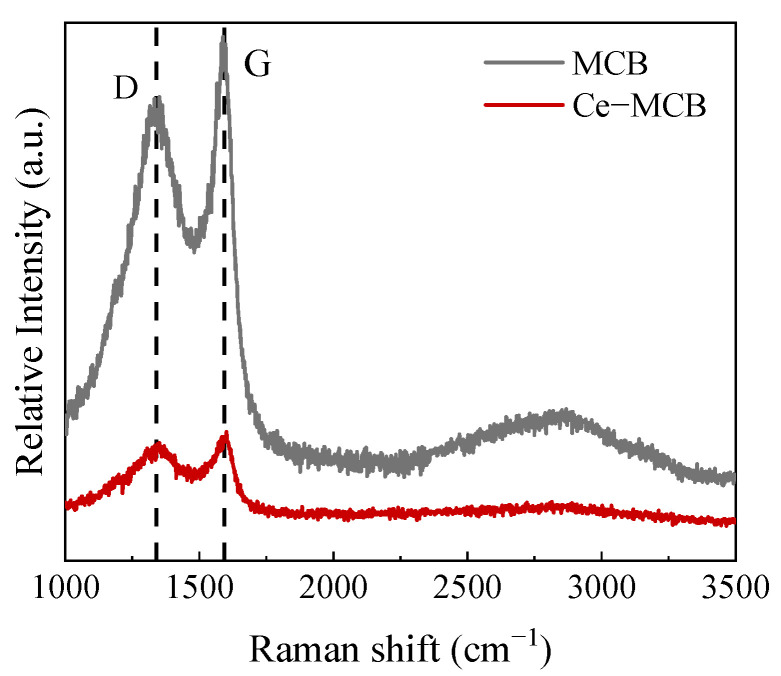
Raman spectra of MCB and Ce-MCB.

The G and D bands measured by RS were widely used to evaluate the microstructure of biochar. The G peak (1580–1600 cm^−1^) was generated by the in-plane tangential vibration of sp2 carbon atoms, while the D peak (1340–1357 cm^−1^) was produced by the oscillation of disordered carbon materials [35,36]. The defect degree of biochar can be indicated by the I_D_/I_G_ value in the Raman spectrum, and the higher the I_D_/I_G_ ratio of the sample, the greater the degree of defect on the surface [37]. From the results in Figure 3, the I_D_/I_G_ value of Ce-MCB (0.875) was larger than that of MCB (0.851), which demonstrated that Ce-MCB possessed higher activity with more abundant defects and functional groups [38]. The increased degree of defects in the biochar modified with Ce(NO_3_)_3_ may be related to the destruction of the original graphitized structure by the doped CeO_2_ and NO_3_^−^. In addition, the attaching metal oxides on the surface of biochar could also act as the active sites of the adsorbent, thus promoting the reaction efficiency [36].

**Figure 4:**
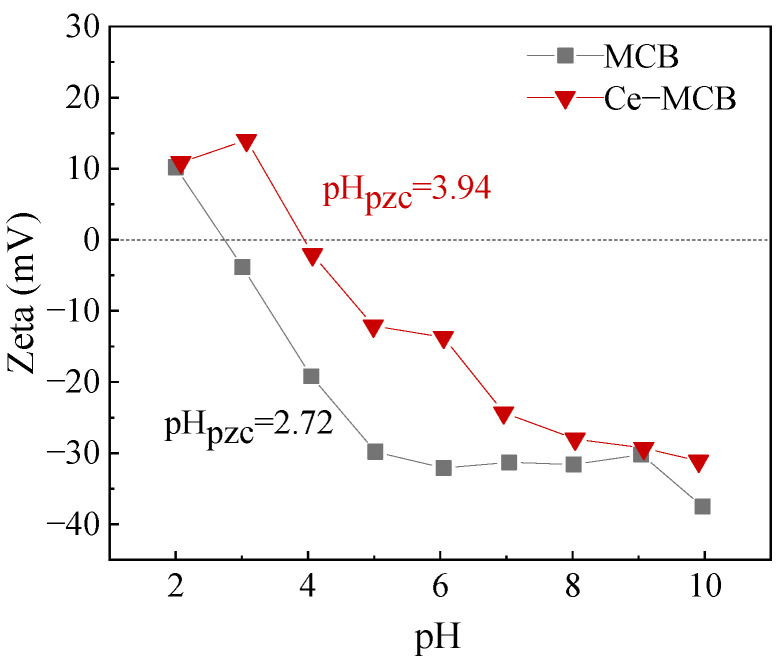
Zeta potential of MCB and Ce-MCB at different solution pH.

The zeta potential is a technique for measuring the surface charge of materials based on the fact that the number of surface charges of biochar varies with the pH value of the solution [39]. When the pH was lower than the point of zero charge (pH_pzc_), the surface of the material was positively charged, which was unfavorable for the absorption of Pb^2+^, due to the electrostatic repulsion. With increasing pH, the carboxylic acid groups and hydroxyl groups were deprotonated. Under the condition that the pH was greater than pH_pzc_, the surface of the biochar was negatively charged, which enhanced the electrostatic attraction [40]. As shown in Figure 4, the point of zero charge of MCB was 2.72, while that of Ce-MCB was increased to 3.94. The zeta potential ranged from 18.3 to −37.5 mV for MCB and from 10.2 to −31.1 mV for Ce-MCB over the pH range of 2–10. It is thus that both biochar surfaces were negatively charged over a wide pH range and that the zeta potential of Ce-MCB was slightly higher than that of MCB. The above result is in accordance with P et al. that Ce-MCB provides more positive charge as compared to MCB, which could be attributed to the greater formation of metal oxide [41].

**Figure 5:**
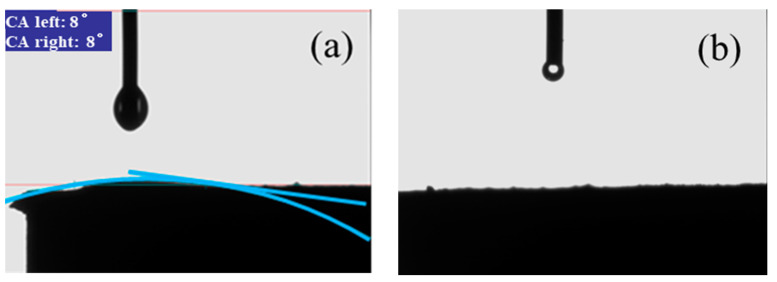
Images of contact angle of (**a**) MCB and (**b**) Ce-MCB in deionized water.

Previous studies have shown a positive correlation between hydrophilic and contact angle for biochar [42]. Biochar had wettability and was capable of absorbing water rapidly as well [43]. The hydrophilic properties of biochar were expressed by measuring the contact angle, as shown in Figure 5. The contact angle of the MCB was 8°, indicating that it was a hydrophilic material. In contrast, Ce-MCB had a contact angle of 0° and was a superhydrophilic material. The results showed that the hydrophilicity of Ce-MCB was higher than that of MCB, which is associated with the preferential orientation of the polar oxygen-containing groups (e.g., hydroxyl groups) enriched on the surface of Ce-MCB toward water [44]. The FTIR test results also prove the presence of these polar groups. Remarkably, the cerium oxide loaded on the Ce-MCB surface was inherently hydrophilic [45].

**Figure 6:**
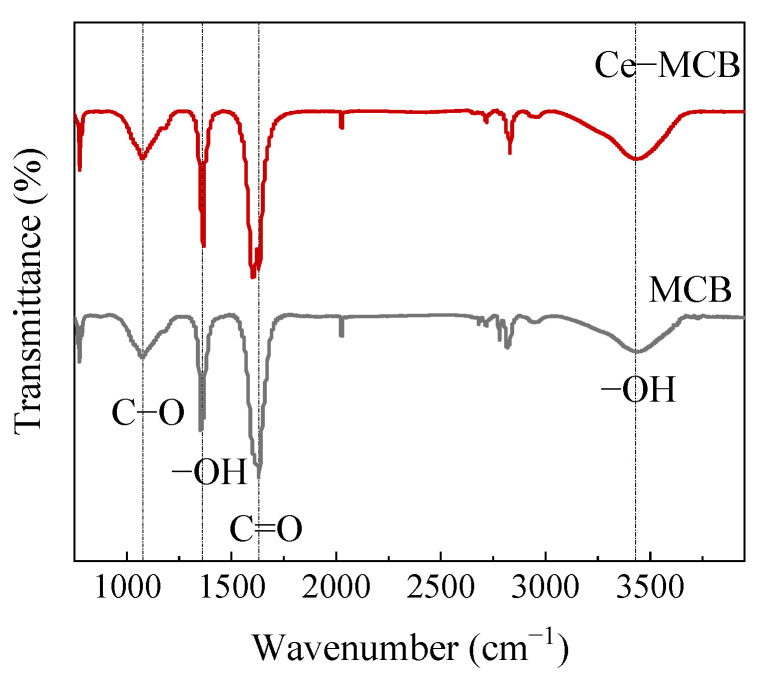
FTIR curves of MCB and Ce-MCB.

FTIR measurements were performed on MCB and Ce-MCB to characterize the functional groups present on the adsorbent surface, which were essential for the absorption process of metal ions. As shown in Figure 6, several peaks were observed in the spectra at about 3440, 1610, 1350, and 1070 cm^−1^. The bands at about 3440 cm^−1^ and 1350 cm^−1^ indicated -OH asymmetric stretching vibration. In the aqueous environment, Ce^3+^ can be used as active sites for the dissociation of H_2_O to form -OH [46]. The bands of 1610 cm^−1^ could be attributable to C=O (COO^−^) stretching and bending vibrations [47]. The 1070 cm^−1^ band corresponded to the vibration of C-O. The peaks of carboxyl and hydroxyl groups were greatly enhanced after Ce(NO_3_)_3_ modification, which facilitates the absorption of heavy metals. The FTIR analysis results were in agreement with RS as well as contact angle results.

**Figure 7:**
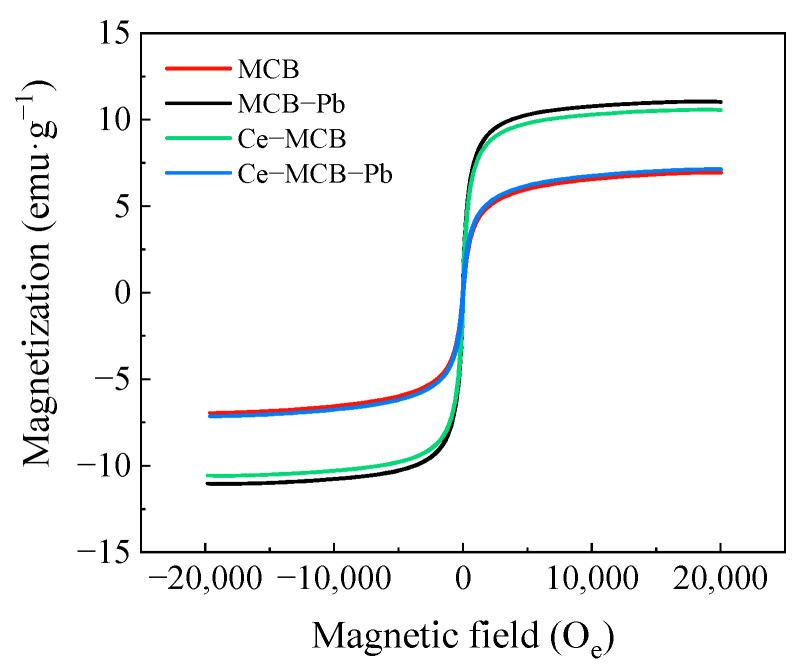
Magnetization curve of MCB and Ce-MCB.

The hysteresis loops of MCB and Ce-MCB were represented as magnetic properties. Figure 7 shows that the saturation magnetization of MCB and Ce-MCB were found to be 11.054 emu·g^−1^ and 7.149 emu·g^−1^, respectively. Although the saturation magnetization intensity of the Ce-MCB was lower than that of MCB, it is sufficient to support an external magnet to magnetically separate it from the solution. The introduction of CeO_2_ decreased the saturation magnetization intensity of biochar. This might be attributed to the oxidation of Ce(NO_3_)_3_, causing the oxidation of iron in the biochar, which can be inferred from the XPS results. The magnetic properties of the biochar remained almost unchanged after absorption of Pb^2+^, indicating that the magnetic properties of the adsorbent were stable.

**Figure 8:**
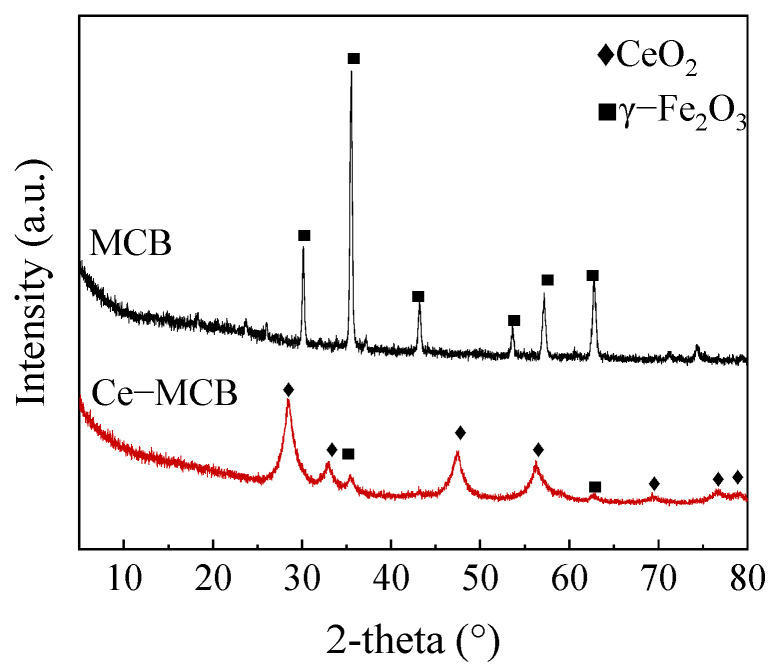
The XRD patterns of MCB and Ce-MCB.

XRD patterns of biochar are presented in Figure 8. The characteristic peaks of the spectrum were located at 30.2°, 35.6°, 43.3°, 53.7°, 57.2°, and 62.9° for γ-Fe_2_O_3_ (JCPDS no. 85-1436) [48]. Additionally, significant diffraction peaks were also observed at 28.5°, 33.1°, 47.5°, 56.3°, 59.1°, 69.4°, 76.7°, and 79.1° in the XRD spectra of Ce-MCB, which corresponded to the (111), (200), (220), (311), (222), (400), (331), and (420) planes of the CeO_2_ (JCPDS card no. 34-0394) [49,50].

**Figure 9:**
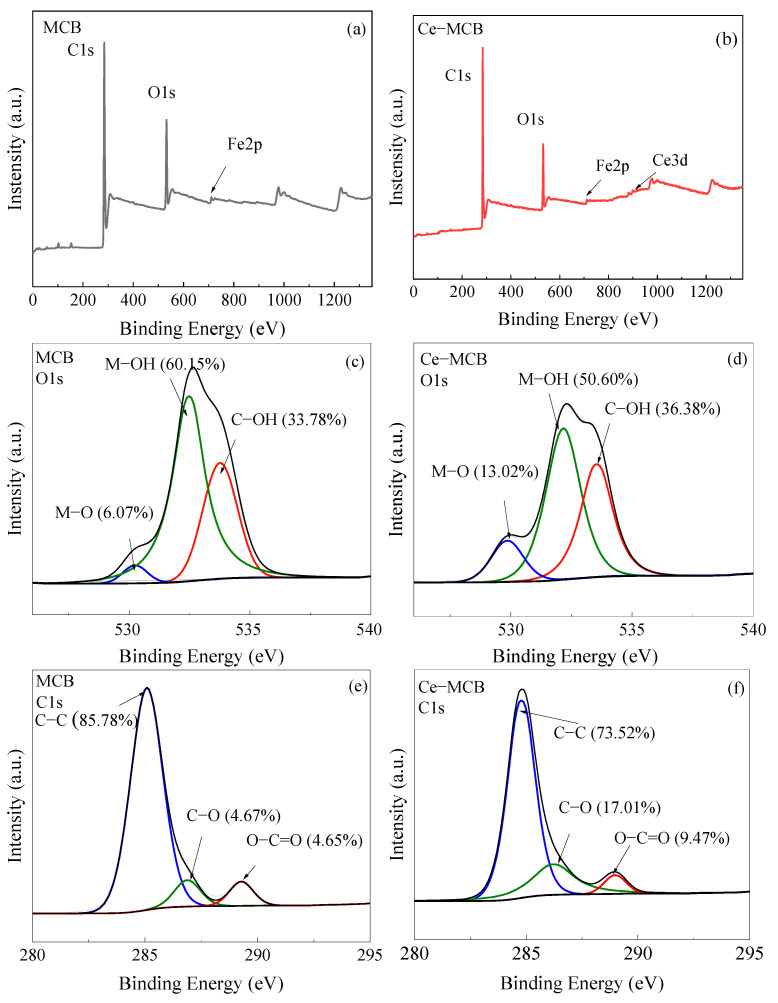

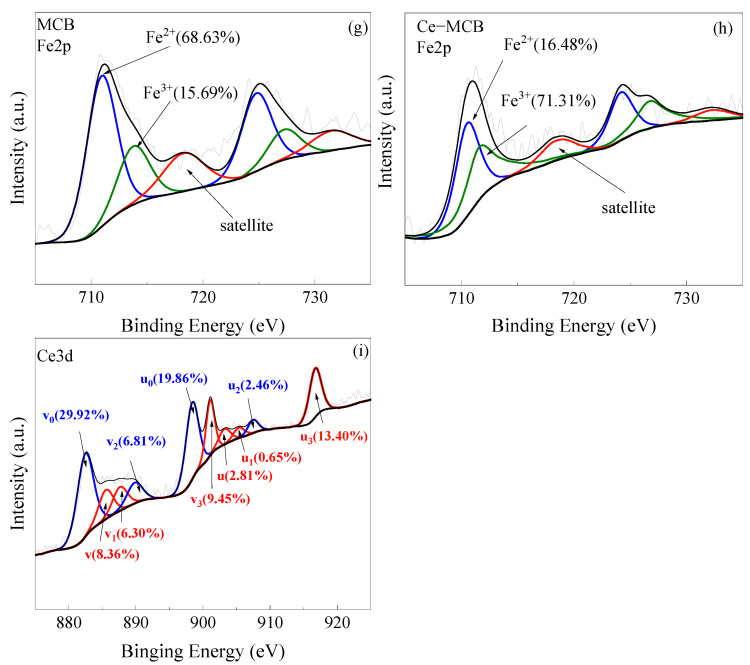
The XPS surveys of MCB and Ce-MCB (**a**,**b**). High-resolution XPS spectra of O1s (**c**,**d**), C 1 s (**e**,**f**), and Fe2p (**g**,**h**). Ce3d spectra of the Ce-MCB (**i**).

The elements surface compositions of each element in magnetic biochar were investigated by XPS, as shown in Figure 9. The C1s spectrum of Ce-MCB can be divided into three parts: C-C (285.08 eV), C-O (286.88 eV), and O-C=O (286.18 eV) [51]. For Ce-MCB, the binding energies of the C-C/C=C, C-O, and O-C=O peaks were shifted to 248.78 eV, 286.18 eV, and 288.98 eV, respectively [52]. Compared with MCB, the percentage of C=C in Ce-MCB decreased while the content of C-O increased. Three peaks of the Ce-MCB O1s spectrum corresponded to M-O (529.88 eV), M-OH (532.18 eV), and C-OH (533.48 eV) [53]. The proportion of M-O increased significantly after modification, indicating the successful loading of rare earth metal oxides. 

In the Fe 2p spectrum, there were two asymmetric peaks at 711.38 eV and 725.18 eV attributed to Fe 2p1/2 and Fe 2p3/2, respectively [54]. Compared with MCB, Ce-MCB had a higher proportion of Fe^3+^ on its surface. The result is consistent with the VSM findings. The Ce oxidation state on the Ce-MCB surface was further studied and the Ce3d_5/2_ and Ce3d_3/2_ spectra are shown in Figure 9i. The spectra were deconvoluted into 10 peaks, where v_0_, v_2_, u_0_, and u_2_ (peaks marked in blue) corresponded to Ce^3+^ and the rest (peaks marked in red) corresponded to Ce^4+^ [55,56].

### 2.2. Pb^2+^ Absorption Experiments

#### 2.2.1. Effect of Solution pH

**Figure 10:**
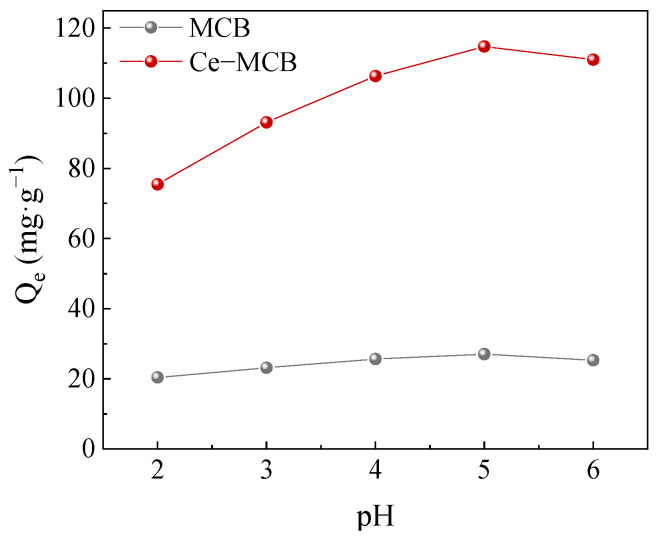
Effect of initial solution pH on Pb^2+^ removal by MCB and Ce-MCB.

The absorption capacity of biochar for heavy metals varies substantially with pH because the presence of Pb^2+^ is influenced by the pH of the solution. Considering that Pb^2+^ generates precipitation, Pb^2+^ begins to precipitate to a pH of solution greater than 5.8 [57]. At solution pH values greater than 6, the Pb^2+^ in solution is readily converted to hydroxide complexes, including but not limited to Pb(OH)^2+^, Pb(OH)_2_, Pb_2_(OH)^3+^, and Pb_3_(OH)_2_^4+^ [58]. When the pH of the solution is around 7, the Pb^2+^ almost completely converts to Pb(OH)_2_(s) [59]. Therefore, the initial pH of the solution was adjusted between ~2 and ~6. Figure 10 reveals that the pH of the initial solution significantly influenced the absorption of Pb^2+^ by magnetic biochar. For both biochars, the absorption capacity of Pb^2+^ increased markedly when the pH increased from 2 to 5. This may be due to the decrease in the concentration of H^+^ ions in the solution and the deprotonation of surface groups, which favored the interaction of biochar with Pb^2+^ [60]. However, between pH 5 and 6, a slight decrease in absorption capacity was observed. The variation might be explained by the presence of Pb^2+^ existing in the form of Pb(OH)^−^, which could precipitate and become the main factor affecting the Pb^2+^ removal. Compared with MCB, the absorption capacity of Ce-MCB was more significantly affected by pH. The result may be explained by the fact that the part of the Ce-MCB surface that played a dominant role in absorption was dissolved in the acidic solution, resulting in a significant decrease in the absorption capacity of Ce-MCB. When the pH increased to 5, the absorption capacity of Pb^2+^ of biochar was highest. Therefore, the subsequent experiments were performed at an optimal solution initial pH of 5.

#### 2.2.2. Absorption Kinetics

**Figure 11:**
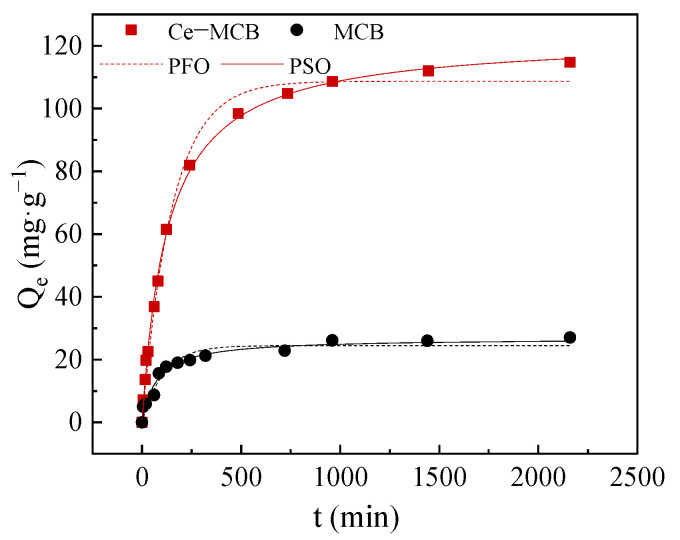
Pseudo-second-order and pseudo-first-order model of biochar to Pb^2+^.

To evaluate absorption kinetics, the effects of contact time on the absorption of Pb^2+^ by MCB and Ce-MCB are illustrated in Figure 11. The parameters of the fitting results for PFO (pseudo-first-order) and PSO (pseudo-second-order) are shown in Table 2. Although the R^2^ value for the PSO (R^2^ = 0.9680, 0.9985) was better than that of the PFO (R^2^ = 0.9429, 0.9928), the PSO also fit the experimental data well. The absorption of Pb^2+^ increased rapidly in the first 200 min and slowed in 200–500 min. After 500 min, the absorption process of the adsorbents reached dynamic equilibrium. The results revealed that chemisorption might be dominated and physical absorption might supplement the absorption process of Pb^2+^ on biochar, and the theoretical maximum absorption values were closer to the values of the actual absorption experiment.

**Figure 12:**
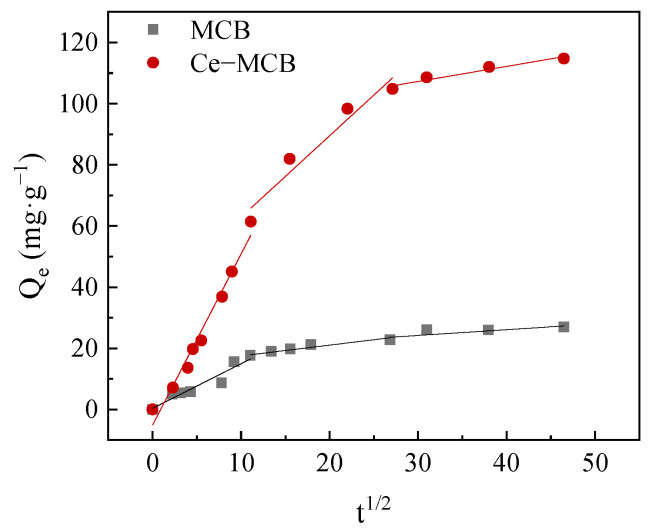
Intra-particle diffusion model of biochar to Pb^2+^.

**Figure 13:**
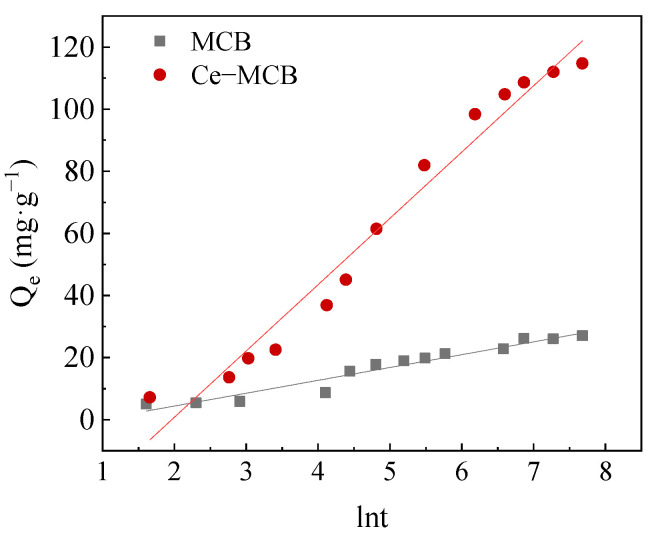
Elovich model of magnetic biochar for Pb^2+^.

To further explore the potential absorption mechanisms of Ce-MCB and MCB, the Elovich and intra-particle diffusion models were also used to formulate the absorption kinetics, and the results are shown in Figure 12 and Figure 13. As shown in Table 3, the intraparticle diffusion model with R^2^ > 0.89 characterizes the adsorption process in stages. The Elovich model with R^2^ > 0.95 (Table 4) also appropriately depicted the absorption process, accounting that the absorption of Pb^2+^ on Ce-MCB was a complex process that might have a linear dependence over a wide time range. Based on the studies of former users, the intra-particle diffusion model was divided into three stages [61,62,63]. In the first stage, Pb^2+^ diffused rapidly on the adsorbent surface and the absorption process occurred on the external surface. With time, the second phase began, the absorption sites were occupied, and the absorption rate gradually decreased until equilibrium. The fitted graphs for three stages deviated from the origin, indicating that chemisorption had an effect on the absorption rate.

#### 2.2.3. Absorption Isotherms

**Figure 14:**
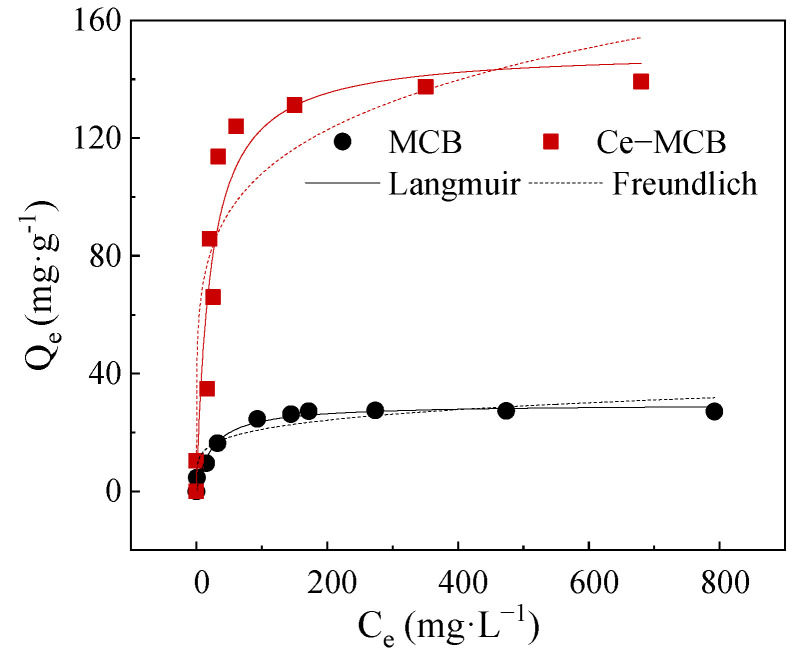
Absorption isotherm of Pb^2+^ adsorbed on biochars.

The Langmuir isotherm equation postulates that the surface of the biochar is homogeneous with the same affinities and that reaction takes place in a monolayer absorption pattern [64]. The Freundlich equation describes the multilayer sorption that occurs on heterogeneous surfaces, in which the availability of absorption sites has different absorption forces [65]. The Langmuir and Freundlich parameters for the absorption of Pb^2+^ are listed in Table 5. The higher correlation coefficient values of the Langmuir model compared to the Freundlich model indicated that the Langmuir model was more suitable for describing the absorption process. As can be seen from Figure 14, the theoretical maximum absorption capacities of MCB and Ce-MCB were 29.61 mg·g^−1^ and 140.83 mg·g^−1^, respectively. The absorption process was attributed to single-molecular-layer absorption and the active sites of biochar were relatively uniformly distributed. In addition, the separation factor (R_L_) allowed for assessing the favorability of the absorption, which is defined by: R_L_ = 1/(1 + K_L_C_0_). The value of this parameter indicates the basic characteristics of the absorption process, which is irreversible (R_L_ = 0), favorable (0 < R_L_ < 1), linear (R_L_ = 1), or unfavorable (R_L_ > 1) [25]. In this study, the value of R_L_ was calculated to be between 0.0079 and 0.023, which confirmed that the absorption process was easy to carry out. The result might be explained by the fact that the high initial concentration of the solution provides the mass transfer driving force for the absorption process.

#### 2.2.4. Reusability Studies

**Figure 15:**
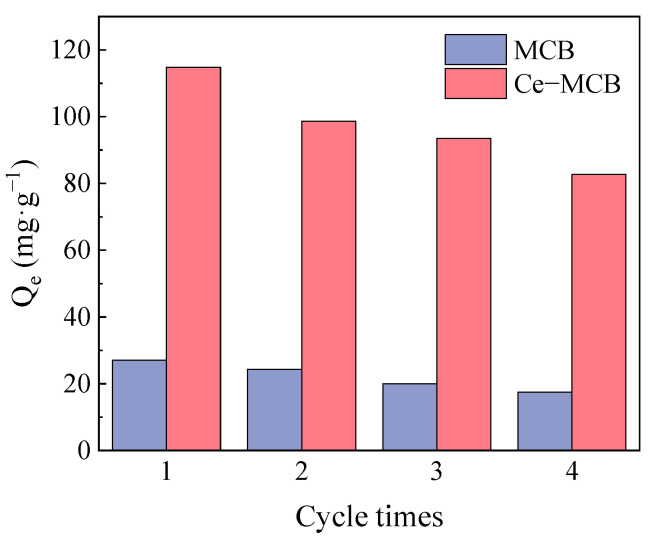
Regeneration of Pb^2+^ absorption on magnetic biochars.

The absorption efficiencies of MCB and Ce-MCB for Pb^2+^ after four cycles were investigated (Figure 15). The results demonstrated that after the fourth cycle, the absorption of Ce-MCB and MCB decreased from 114.74 mg·g^−1^ to 82.63 mg·g^−1^ and 27.01 mg·g^−1^ to 17.47 mg·g^−1^, respectively. These reductions in absorption capacity were mainly due to the partial loss of minerals from the adsorbent surface during the desorption process.

#### 2.2.5. Effect of Coexisting Alkali Metal Ions

**Figure 16:**
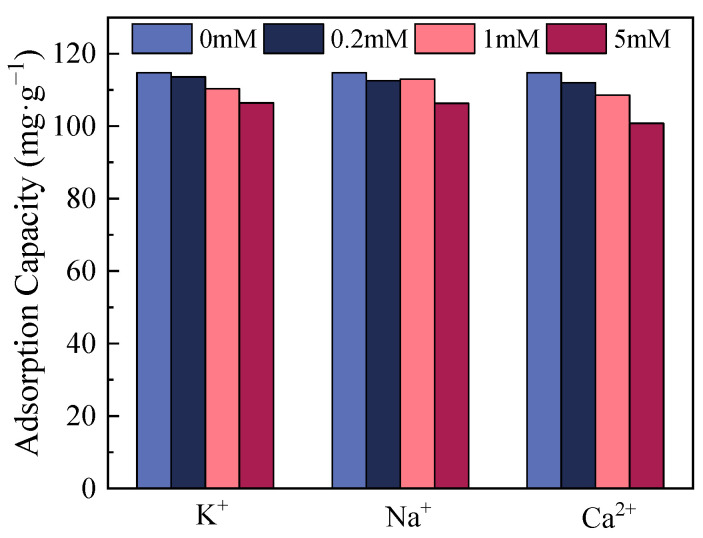
Effects of different concentration of coexisting cations on Pb^2+^ absorption on to Ce-MCB.

There are common metal cations (Na^+^, K^+^, and Mg^2+^) in industrial wastewater, which might interfere with the removal of Pb^2+^ [66]. The effects of alkali metal ions (K^+^, Na^+^, and Ca^2+^) and strength on Pb^2+^ absorption by Ce-MCB were investigated. As displayed in Figure 16, the presence of K^+^ and Na^+^ had little effect on the sorption capacity of Pb^2+^. This may be ascribed to the low charge density of K^+^ and Na^+^, which did not compete with Pb^2+^ for absorption sites [67]. However, the absorption capacity of Ce-MCB for Pb^2+^ was significantly affected by Ca^2+^, while in 5 mM, we can conclude that Ca^2+^ had a stronger inhibitory effect on the absorption process of Pb^2+^ compared to K^+^ and Na^+^. On the one hand, the absorption of Pb^2+^ decreased by more than 10%, and the presence of Ca^2+^ formed a competitive absorption with Pb^2+^, occupying part of the absorption site of Pb^2+^. On the other hand, the presence of Ca^2+^ led to the aggregation of CeO_2_ nanoparticles [68], resulting in the compression of the electric double layer and the formation of a dense structure, which reduces the effective available area for biochar absorption and decreases the active sites.

Table 6 shows the maximum absorption capacity (Q_m_) of Ce-MCB for Pb^2+^ in comparison with those gained in other investigations. Despite the different experimental conditions used, the comparison of Q_m_ values was useful because the absorption capacity was the criterion that provided the performance of Ce-MCB relative to other materials. Among the listed materials, the Ce-MCB showed excellent performance based on Pb^2+^ removal efficiency.

### 2.3. Absorption Mechanisms

#### 2.3.1. FTIR Analysis

**Figure 17:**
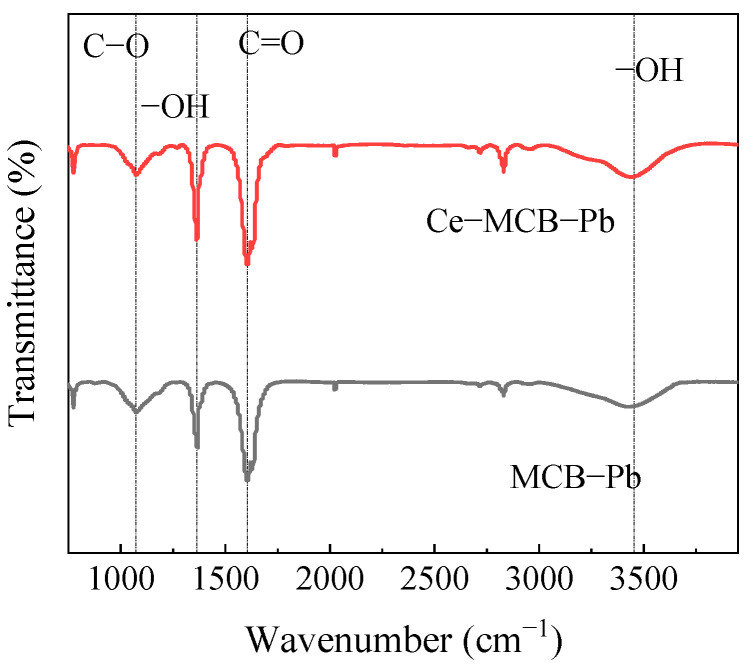
FTIR spectrum of biochar after Pb^2+^ absorption.

The hydroxyl and carboxyl functional group on the surface perform an essential role in the Pb absorption process. In the FTIR spectra of biochar after absorption (see Figure 17), the peak of Ce-MCB biochar at 3440 cm^−1^ (-OH bending vibration) was significantly attenuated. In addition, the peak at 1382 cm^−1^ (O-H bending vibration) shifted mildly to 1375 cm^−1^. These changes indicated that -OH was involved in the absorption process of Pb^2+^ and formed a monodentate complex on the biochar surface [75,76].The absorption peak located at 1610 cm^−1^ was weakened, indicating that the carboxyl group was also involved in the absorption of Pb^2+^. MCB-Pb showed a similar variation to Ce-MCB-Pb.

#### 2.3.2. XRD Analysis

**Figure 18:**
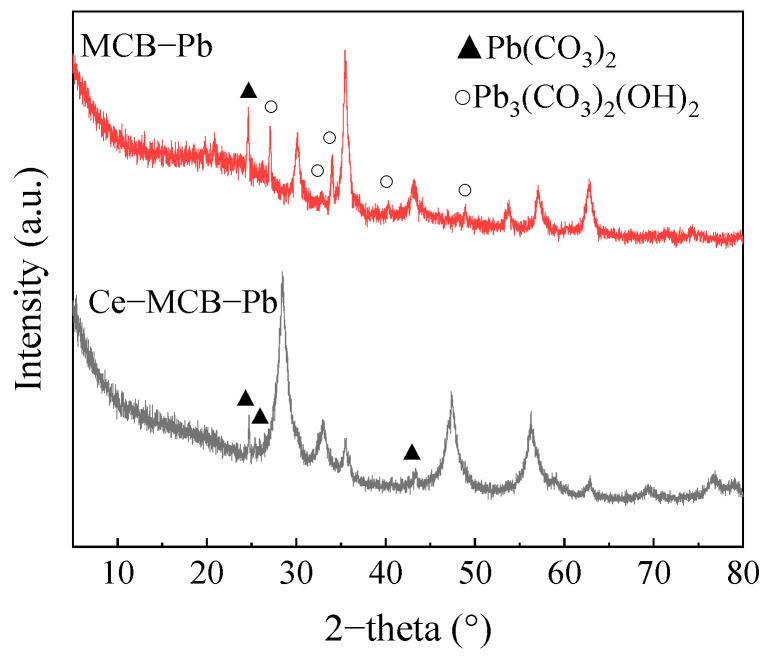
XRD patterns of MCB-Pb and Ce-MCB-Pb.

The XRD analysis of MCB and Ce-MCB after Pb^2+^ absorption is shown in Figure 18. After the absorption of Pb^2+^, new diffraction peaks were observed on the surface of biochar. The emerging peaks corresponded to the diffraction peaks of PbCO_3_ and Pb_3_(CO_3_)_2_(OH)_2_, showing the chemical reactions that occurred during the absorption of Pb^2+^ and the formation of PbCO_3_ and Pb_3_(CO_3_)_2_(OH)_2_ crystals. Therefore, it could be determined that the precipitation formed on the surface of biochar was one of the mechanisms of Pb^2+^ removal, which may be caused by the co-precipitation reaction between Pb^2+^ and minerals (such as CO_3_^2−^ and HCO_3_^−^) [77].

#### 2.3.3. XPS Analysis

**Figure 19:**
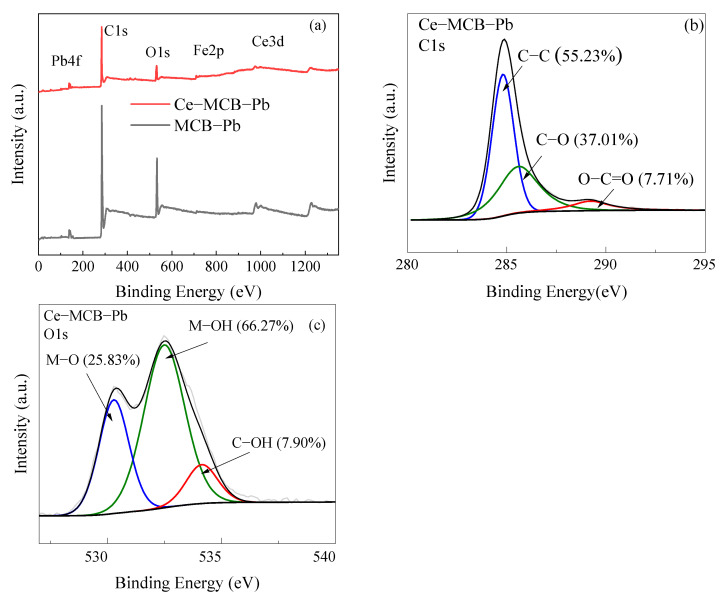
The XPS surveys of MCB-Pb and Ce-MCB-Pb (**a**) C 1 s (**b**) and O1s (**c**) spectra for the Ce-MCB-Pb.

XPS was also used to analyze the absorption mechanism. XPS surveys of biochar after absorption are presented in Figure 19. The binding energies and ratios of the three peaks in the O1s spectra changed significantly. After absorption of Pb^2+^, the C-C peak area ratio of Ce-MCB decreased from 77.52% to 55.23%, suggesting that π–π on the adsorbent surface had cation–π interaction with Pb^2+^ [78]. The O1s spectrum of after absorption showed that the proportion of both M-O and M-OH was increased, indicating the formation of a monodentate (-O-Pb-OH) and bidentate complex (-O-Pb-O). C-OH decreased from 36.38% to 7.90%, indicating that the absorption consumed -OH and there was monodentate chelation. These values emphasized that M-O, M-OH, and C-OH were the critical groups for Pb^2+^ absorption.

#### 2.3.4. Relative Distributions of Absorption Mechanisms

**Figure 20:**
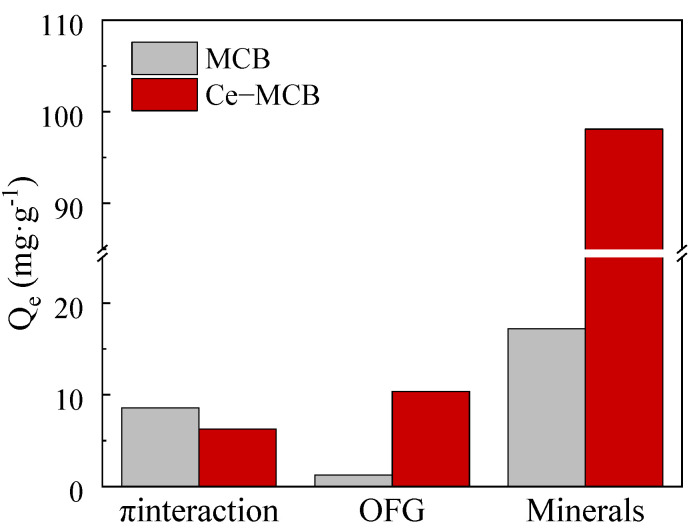
Quantitative study on the mechanism of Pb^2+^ absorption by MCB and Ce-MCB.

The removal of Pb^2+^ was ascribed to three fractions: Q_m_, Q_π_, and Q_f_. Compared with MCB, the Q_m_ and Q_f_ values of Ce-MCB increased significantly, while the values of Q_π_ remained almost unchanged. As shown in Figure 20, the value of the oxygen functional groups contribution increased from 1.24 mg·g^−1^ to 10.37 mg·g^−1^. The increase in Q_f_ was due to the Ce(NO_3_)_3_ treatment, which introduced more oxygen-containing functional groups and enhanced the absorption properties of the functional groups. Due to the high adsorption capacity of minerals to heavy metals, the increase in the proportion of minerals caused by the loading of CeO_2_ on the surface of biochar is also an important reason for the improvement of adsorption capacity. Compared with MCB, the Q_m_ value of Ce-MCB was increased by 80.899 mg·g^−1^. However, the Q_π_ values of MCB and Ce-MCB were almost similar, indicating that the CeO_2_-doped was free of a significant effect on Q_π_ [79].

## 3. Materials and Methods

### 3.1. Materials

Reagents including ferric nitrate hydrate (Fe(NO_3_)_3_·9H_2_O), cerium nitrate hydrate (Ce(NO_3_)_3_·9H_2_O), lead nitrate (Pb(NO_3_)_2_), potassium chloride (KCl), calcium chloride (CaCl_2_), sodium chloride (NaCl), sodium hydroxide (NaOH), HCl, HNO_3_, and absolute ethyl alcohol were purchased from Shanghai Maclean Co., Ltd. (Shanghai, China).

### 3.2. Preparation of Magnetic Biochar

The coconut skin fiber was purchased from an orchard site in Haikou, Hainan province. The ingredients were washed with deionized water to remove the impurities, and subsequently dried in an oven at 105 °C to a constant weight. After crushing into powder and passing through a 20-mesh sieve, the coconut coir was stored in a plastic zip lock bag in the laboratory.

Coconut skin fiber (CS) of 11.22 g and Fe(NO_3_)_3_·9H_2_O of 4.04 g were mixed with 100 mL of ethanol in a magnetic stirrer for 24 h. For Ce(NO_3_)_3_/Fe(NO_3_)_3_ co-modification, the coconut skin of 11.22 g was mixed with 4.04 g of Fe(NO_3_)_3_·9H_2_O and 4.34 g of Ce(NO_3_)_3_·6H_2_O and stirred for 24 h. The suspensions were mixed in ultrasound for 1 hour and then dried in oven at 65 °C. The Fe(NO_3_)_3_-loaded CS (MCS) and Ce(NO_3_)_3_/Fe(NO_3_)_3_-loaded CS (Ce-MCS) were dried and packed in a crucible, which was placed in a tube furnace and calcined to 600 °C with a ramp of 10 °C min^−1^ and under flowing N_2_ for 2 h. The obtained biochar was named as MCB and Ce-MCB.

### 3.3. Characterization of the Biochars

Scanning electron microscopy (SEM, Hitachi Limited SU-70, Tokyo, Japan) was used to be examined the appearance and morphology of biochar samples. The specific surface areas (SSAs), total pore volume (V_t_), and porosity were measured by an N_2_ absorption and desorption analyzer (Quantachrome, QI3, Boynton Beach, FL, USA) at 77 K. The surface organic functional groups were characterized using Fourier transform infrared spectroscopy (FTIR, Thermo Fisher Nicolet iS50, Waltham, MA, USA). Raman spectroscopy (Raman, HORIBA Jobin Yvon LabRAM HR800, Montpellier, French) recorded the degree of defects and graphitization. X-ray diffraction (XRD, PANalytical X’Pert Pro MPD, Heracles Almelo, The Netherlands) was used to characterize the crystalline structure of biochar. The surface composition and chemical valence of biochar was analyzed by X-ray photoelectron spectroscopy (XPS, Scientific Thermo Fisher K-Alpha, Waltham, MA, USA). Hydrophilic properties were calculated using the contact angle gauges. The zeta potential of biochar was determined using the laser particle sizer (Zeta, Malvern nano ZS & Mastersizer, Malvern, UK). A vibrating sample magnetometer (VSM, Lake Shore Lake Shore 7404, Westerville, OH, USA) was used to detect the magnetization of biochar.

### 3.4. Absorption Experiment

The Pb(NO_3_)_3_ of 1.598 g was dissolved in deionized water to configure a solution with a concentration of 1 g L^−1^. The solution was diluted with ultrapure water to the required concentration and the initial pH (LICHEN pH-100, Changsha, China) of the solution was controlled at 5 by 1 M NaOH and 1 M HCl. The experimental conditions were adjusted to 5 ± 0.2 and the ion concentration was 200 mg·L^−1^ (the absorption amount was close to saturation at the ion concentration of 200 mg·L^−1^).

Absorption kinetics of Pb^2+^ onto 0.3 g of biochar were placed in conical flasks containing 300 mL of aqueous solution. In a typical reaction, the flask was shaken in a water bath shaker with a fixed speed of 200 rpm. At predetermined time intervals, 2 mL of solution was collected and its residual Pb^2+^ concentration was detected by ICP-OES.

Absorption isotherms of Pb^2+^ onto 10 mg of biochar were added to the centrifugal tubes with 10 mL of Pb^2+^ solution where the concentration was from 0 to 1000 mg L^−1^. The tubes were shaken at 200 rpm in a water bath shaker for 48 h. After the absorption equilibrium, all samples were filtered, and the heavy metal concentrations were determined by the same method.

To investigate the reusability of magnetic biochar in a real environment, the regeneration was conducted. In each cycle, the Pb^2+^ adsorbed MCB or Ce-MCB was magnetically separated from the absorption mixture and then transferred to another conical flask containing 300 mL of CH_3_COONa aqueous (1 mol·L^−1^ for a 2 h desorption). The desorption–absorption experiment was repeated for 4 cycles.

Investigating the influence of pH value on absorption behavior, 10 mL Pb^2+^ (200 mg L^−1^) solutions were added in centrifugal tubes. The initial pH of the solution was adjusted to 2.0–6.0 by droplets of 0.1 M HCl and 0.1 M NaOH. In the test for the effect of co-existing alkali metal ions, the solution with different concentrations of K^+^/Na^+^/Ca^2+^ was added to the Pb^2+^ (200 mg L^−1^). An amount of 10 mg of biochar was added to the solution and the tubes were shaken at 200 rpm for 48 h. The Pb^2+^ concentration in all samples was detected by ICP-OES.

### 3.5. Model Fitting

The biochar equilibrium absorption amount *q_t_* (mg g^−1^) of Pb^2+^ was calculated by Equation (1):

(1) $$q_{t}=\frac{\left(C_{0}-C_{t}\right)\;V}{M}$$

where *C*_0_ (mg L^−1^) represents the initial concentration of Pb^2+^; *C_t_* (mg L^−1^) is the concentration of Pb^2+^ in the solution at time *t* (h); *V* (mL) is the volume of absorption solution; *M* (mg) is the mass of biochar; *q_t_* (mg g^−1^) is the Pb^2+^-adsorbed amount at a time *t* (min).

To explain the details of absorption kinetics, the pseudo-first-order kinetic model (PFO), pseudo-second-order kinetic model (PSO), Elovich model, and intra-particle diffusion model were used to describe the absorption kinetics, and the results of the fits are shown below [51,80].

(2) $$\mathrm{PFO}:\;q_{t}=q_{e}\left(1-e^{-k_{1}t}\right)$$

(3) $$\mathrm{PSO}:\;q_{t}=\frac{k_{2}q_{e}^{2}t}{1+k_{2}q_{e}t}$$

(4) $$\mathrm{Elovich}:\;q_{t}=\frac{1}{\beta }\ln\left(\alpha \beta \right)+\frac{1}{\beta }lnt$$

(5) $$\mathrm{Intra}-\mathrm{particle}\;\mathrm{diffusion}:\;q_{t}=k_{id}t^{1/2}$$

where *k*_1_ is the absorption rate constant of PFO; *k*_2_ represents the equilibrium rate constant of PSO; *q_e_* (mg g^−1^) is the Pb^2+^ adsorbed amount at equilibrium; *α* is the initial absorption rate constant; *β* is the desorption rate constant; *k_id_* (mg·g^−1^·min^−1/2^) is the intra-particle diffusion rate constant.

The models of Langmuir and Freundlich were used to describe the absorption isotherm [81]:

(6) $$\mathrm{Langmuir}:\;q_{e}=\frac{Q_{0}K_{L}C_{e}}{1+K_{L}C_{e}}$$

(7) $$\mathrm{Freundlich}:\;q_{e}=K_{F}C_{e}^{1/n}$$

where *K_F_* and *K_L_* represent the absorption rate constants of the Langmuir and Freundlich equations, respectively; *Q*_0_ is the Langmuir static theoretical absorption capacity; *C_e_* is the concentration of equilibrium adsorbent in the aqueous phases; *n* is the isothermal constant of the Freundlich isotherm curvature.

### 3.6. Quantitative Analysis of the Different Absorption Mechanisms

The absorption mechanism of Pb^2+^ was composed of three parts: surface complexation of functional groups (*Q_f_*), mineral precipitation (*Q_m_*), and coordination of metal–π–electron (Pb^2+^-π) interaction (*Q_π_*).

The biochar was acid-washed and demineralized for removal, at which point there was no change in the functional groups on the surface of the biochar. The reduced absorption capacity during the de-ashed biochar was the contribution of the minerals. The mechanism of absorption following de-ashed biochar includes oxygen functional groups and π interaction. During the contribution of oxygen functional groups, hydroxyl and carboxyl groups released H^+^ into solution by the reaction. Therefore, the pH of the solution was measured before and after absorption of demineralized biochar, the amount of H^+^ released was calculated, and, accordingly, the amount of Pb^2+^ adsorbed by the functional group complexation (*Q_f_*) was calculated. The remainder of de-ashed biochar was considered to be π–electron interaction (*Q_π_*), as shown in Equation (9). [57]

*Q_m_* = *Q_e_* − *Q_a_* (8)

*Q_π_* = *Q_a_* − *Q_f_* (9)

where *Q_a_* is the absorption capacity of biochar after HCl washing, *Q_m_* is the absorption by mineral precipitation, *Q_e_* is the total absorption, *Q_π_* is the absorption by π–electron interaction, and *Q_f_* is the absorption by complexation of oxygen-containing functional groups.

## 4. Conclusions

The combination of magnetic coconut coir biochar and CeO_2_ has proven to be a valuable option for creating high-capacity and reusable adsorbents. FTIR, XRD, and XPS analysis demonstrated that Ce(NO_3_)_3_ modification greatly increased the content of the inorganic mineral fraction and oxygen-containing functional groups of the biochar, which provided more active sites for the adsorption of Pb^2+^. The VSM results showed that the Ce-MCB had sufficient magnetic properties to readily achieve magnetic separation. The modified adsorbent displayed enhanced adsorption capacity for Pb^2+^ with a maximum theoretical adsorption capacity of 140.83 mg·g^−1^, which was 4.8 times higher than that of MCB. Ce-MCB exhibited good reusability characteristics in sequential cycling experiments, maintaining a high adsorption capacity after four cycles still. Additionally, a comprehensive characterization of the Ce-MCB after Pb adsorption revealed that the primary absorption mechanism of Ce-MCB was the acidic oxygen-containing functional group complexation (*Q_m_* = 98.11 mg·g^−1^), π–electron interaction (*Q_π_* = 6.26 mg·g^−1^), and inorganic precipitation (*Q_f_* = 10.37 mg·g^−1^). These results demonstrate the outstanding performance of Ce-MCB in biomass recycling for environmental remediation.

## Figures and Tables

**Table 1:** Pore structural parameters of biochar.

Samples	Specific Surface Area (m^2^·g^−1^)	Pore Volume (cm^3^·g^−1^)	Average Pore Size (nm)
MCB	234.35	0.030	4.99
Ce-MCB	246.32	0.070	5.14

**Table 2:** The fitting parameters of pseudo second-order and pseudo first-order.

Adsorbents	PFO			PSO		
	Q_e_	K_1_	R^2^	Q_e_	K_2_	R^2^
MCB	24.4702	0.00928	0.9429	26.4702	4.9319 × 10^−4^	0.9680
Ce-MCB	108.7895	0.00657	0.9928	122.7613	6.3576 × 10^−5^	0.9985

**Table 3:** The fitting parameters of intra-particle diffusion.

Adsorbents	Intra-Particle Diffusion		
	R_1_^2^	R_2_^2^	R_3_^2^
MCB	0.9312	0.9170	0.8942
Ce-MCB	0.9714	0.9430	0.9492

**Table 4:** The fitting parameters of Elovich.

Adsorbents	Elovich		
	α (mg·(g·min)^−1^)	β (g·mg^−1^)	R^2^
MCB	−3.8882	4.1366	0.9528
Ce-MCB	−41.9298	21.3492	0.9696

**Table 5:** The fitting parameters of absorption kinetics.

Adsorbents	Langmuir			Freundlich		
	K_L_	Q_m_	R^2^	n	K_F_	R^2^
MCB	0.04083	29.6060	0.9741	5.0132	8.4063	0.8975
Ce-MCB	0.1253	140.8337	0.9928	5.3815	45.8808	0.8445

**Table 6:** Results of the study on the absorption capacity of adsorbed Pb^2+^.

Materials	Q_m_ (mg·g^−1^)	Concentration (mg·L^−1^)	Activation	Reference
Coconut skin fiber	140.83	200	Fe(NO_3_)_3_, Ce(NO_3_)_2_	This work
	29.61	200	Fe(NO_3_)_3_	
Coconut shell	26.14	/	/	[69]
	49.92	/	HPO_3_	
Coconut shell	66.86	/	Ultraviolet (UV)	[70]
Waste coconut buttons	92.72	300	/	[71]
Pine leaves	31.04	/	/	[72]
Precursor fibers	22.95	100	Fe_3_O_4_	[73]
Raw date leaves and stalks	61.25	25	HNO_3_, DDCFeCl_2_, FeCl_3_	[74]

## Data Availability

No applicable.

## References

[1] Xiao H., Shahab A., Li J., Xi B., Sun X., He H., Yu G. (2019). Distribution, ecological risk assessment and source identification of heavy metals in surface sediments of Huixian karst wetland, China. Ecotoxicol. Environ. Saf..

[2] Tume P., Barrueto K., Olguin M., Torres J., Cifuentes J., Ferraro F.X., Roca N., Bech J., Cornejo O. (2020). The influence of the industrial area on the pollution outside its borders: A case study from Quintero and Puchuncavi districts, Chile. Environ. Geochem. Health.

[3] Abdel-Halim S.H., Shehata A.M.A., El-Shahat M.F. (2003). Removal of lead ions from industrial waste water by different types of natural materials. Water Res..

[4] Jarup L. (2003). Hazards of heavy metal contamination. Br. Med. Bull..

[5] Tan Y., Wan X., Zhou T., Wang L., Yin X., Ma A., Wang N. (2022). Novel Zn-Fe engineered kiwi branch biochar for the removal of Pb(II) from aqueous solution. J. Hazard. Mater..

[6] Dong J., Shen L., Shan S., Liu W., Qi Z., Liu C., Gao X. (2022). Optimizing magnetic functionalization conditions for efficient preparation of magnetic biochar and adsorption of Pb(II) from aqueous solution. Sci. Total Environ..

[7] Amrulloh H., Kurniawan Y.S., Ichsan C., Jelita J., Simanjuntak W., Situmeang R.T.M., Krisbiantoro P.A. (2021). Highly efficient removal of Pb(II) and Cd(II) ions using magnesium hydroxide nanostructure prepared from seawater bittern by electrochemical method. Colloids Surf. A Physicochem. Eng. Asp..

[8] Jagaba A.H., Kutty S.R.M., Hayder G., Baloo L., Ghaleb A.A.S., Lawal I.M., Abubakar S., Al-dhawi B.N.S., Almahbashi N.M.Y., Umaru I. (2021). Degradation of Cd, Cu, Fe, Mn, Pb and Zn by Moringa-oleifera, zeolite, ferric-chloride, chitosan and alum in an industrial effluent. Ain Shams Eng. J..

[9] Bora A.J., Dutta R.K. (2019). Removal of metals (Pb, Cd, Cu, Cr, Ni, and Co) from drinking water by oxidation-coagulation-absorption at optimized pH. J. Water Process Eng..

[10] Fang Y., Ren G., Ma Y., Wang C., Li M., Pang X., Pan Q., Li J. (2022). Adsorption and reutilization of Pb(II) based on acid-resistant metal-organic gel. Sep. Purif. Technol..

[11] Fu F., Wang Q. (2011). Removal of heavy metal ions from wastewaters: A review. J. Environ. Manag..

[12] He W., Zheng S., Chen X., Lu D., Zeng Z. (2022). Alkaline aging significantly affects Mn(II) adsorption capacity of polypropylene microplastics in water environments: Critical roles of natural organic matter and colloidal particles. J. Hazard. Mater..

[13] Mu R., Liu B., Chen X., Wang N., Yang J. (2020). Hydrogel adsorbent in industrial wastewater treatment and ecological environment protection. Environ. Technol. Innov..

[14] Orucoglu E., Tournassat C., Robinet J.-C., Made B., Lundy M. (2018). From experimental variability to the sorption related retention parameters necessary for performance assessment models for nuclear waste disposal systems: The example of Pb adsorption on clay minerals. Appl. Clay Sci..

[15] Mercado-Borrayo B.M., Contreras R., Sanchez A., Font X., Schouwenaars R., Ramirez-Zamora R.M. (2020). Optimisation of the removal conditions for heavy metals from water: A comparison between steel furnace slag and CeO2 nanoparticles. Arab. J. Chem..

[16] Rajendran S., Priya A.K., Kumar P.S., Hoang T.K.A., Sekar K., Chong K.Y., Khoo K.S., Ng H.S., Show P.L. (2022). A critical and recent developments on adsorption technique for removal of heavy metals from wastewater-A review. Chemosphere.

[17] Wang Q., Wang B., Lee X., Lehmann J., Gao B. (2018). Sorption and desorption of Pb(II) to biochar as affected by oxidation and pH. Sci. Total Environ..

[18] Wu W., Li J., Niazi N.K., Muller K., Chu Y., Zhang L., Yuan G., Lu K., Song Z., Wang H. (2016). Influence of pyrolysis temperature on lead immobilization by chemically modified coconut fiber-derived biochars in aqueous environments. Environ. Sci. Pollut. Res..

[19] Li J., Wang S.-L., Zheng L., Chen D., Wu Z., Xie Y., Wu W., Niazi N.K., Ok Y.S., Rinklebe J. (2019). Sorption of lead in soil amended with coconut fiber biochar: Geochemical and spectroscopic investigations. Geoderma.

[20] Wang S.Y., Tang Y.K., Chen C., Wu J.T., Huang Z.N., Mo Y.Y., Zhang K.X., Chen J.B. (2015). Regeneration of magnetic biochar derived from eucalyptus leaf residue for lead(II) removal. Bioresour. Technol..

[21] Shi Q., Zhang H., Shahab A., Zeng H., Zeng H., Bacha A.-U.-R., Nabi I., Siddique J., Ullah H. (2021). Efficient performance of magnesium oxide loaded biochar for the significant removal of Pb^2+^ and Cd^2+^ from aqueous solution. Ecotoxicol. Environ. Saf..

[22] Yin G., Bi L., Song X., Luo H., Ji P., Lin Q., Liu Q., Tang G. (2019). Adsorption of Cd(II) from aqueous solution by Pennisetum sp. straw biochars derived from different modification methods. Environ. Sci. Pollut. Res..

[23] Tong S., Deng H., Wang L., Huang T., Liu S., Wang J. (2018). Multi-functional nanohybrid of ultrathin molybdenum disulfide nanosheets decorated with cerium oxide nanoparticles for preferential uptake of lead (II) ions. Chem. Eng. J..

[24] Hua M., Zhang S., Pan B., Zhang W., Lv L., Zhang Q. (2012). Heavy metal removal from water/wastewater by nanosized metal oxides: A review. J. Hazard. Mater..

[25] Meepho M., Sirimongkol W., Ayawanna J. (2018). Samaria-doped ceria nanopowders for heavy metal removal from aqueous solution. Mater. Chem. Phys..

[26] Recillas S., Garcia A., Gonzalez E., Casals E., Puntes V., Sanchez A., Font X. (2011). Use of CeO_2_, TiO_2_ and Fe_3_O_4_ nanoparticles for the removal of lead from water Toxicity of nanoparticles and derived compounds. Desalination.

[27] Patwardhan P.R., Satrio J.A., Brown R.C., Shanks B.H. (2010). Influence of inorganic salts on the primary pyrolysis products of cellulose. Bioresour. Technol..

[28] Mian M.M., Liu G.J., Yousaf B., Fu B., Ullah H., Ali M.U., Abbas Q., Munir M.A.M., Ruijia L. (2018). Simultaneous functionalization and magnetization of biochar via NH3 ambiance pyrolysis for efficient removal of Cr (VI). Chemosphere.

[29] Singh V., Srivastava V.C. (2020). Self-engineered iron oxide nanoparticle incorporated on mesoporous biochar derived from textile mill sludge for the removal of an emerging pharmaceutical pollutant. Environ. Pollut..

[30] Mei Y.L., Xu J., Zhang Y., Li B., Fan S.S., Xu H.C. (2021). Effect of Fe-N modification on the properties of biochars and their adsorption behavior on tetracycline removal from aqueous solution. Bioresour. Technol..

[31] Ren J., Li N., Li L., An J.K., Zhao L., Ren N.Q. (2015). Granulation and ferric oxides loading enable biochar derived from cotton stalk to remove phosphate from water. Bioresour. Technol..

[32] Gupta A.D., Giri B.S., Rene E.R., Chaturvedi P., Goswami M., Singh H. (2021). Batch and continuous reactor studies for the adsorption of As(III) from wastewater using a hybrid biochar loaded with transition metal oxides: Kinetics and mass transfer analysis. Environ. Eng. Res..

[33] Kikuchi Y., Qian Q.R., Machida M., Tatsumoto H. (2006). Effect of ZnO loading to activated carbon on Pb(II) adsorption from aqueous solution. CARBON.

[34] Cao Y., Zhang H., Liu K., Zhang Q., Chen K.-J. (2019). Biowaste-Derived Bimetallic Ru-MoOx Catalyst for the Direct Hydrogenation of Furfural to Tetrahydrofurfuryl Alcohol. Acs Sustain. Chem. Eng..

[35] Schoenfelder R., Aviles F., Knupfer M., Azamar-Barrios J.A., Gonzalez-Chi P.I., Ruemmeli M.H. (2014). Influence of architecture on the Raman spectra of acid-treated carbon nanostructures. J. Exp. Nanosci..

[36] Bao D., Li Z., Tang R., Wan C., Zhang C., Tan X., Liu X. (2021). Metal-modified sludge-based biochar enhance catalytic capacity: Characteristics and mechanism. J. Environ. Manag..

[37] Huang S., Liang Q., Geng J., Luo H., Wei Q. (2019). Sulfurized biochar prepared by simplified technic with superior adsorption property towards aqueous Hg(II) and adsorption mechanisms. Mater. Chem. Phys..

[38] Fan J., Li Y., Yu H., Li Y., Yuan Q., Xiao H., Li F., Pan B. (2020). Using sewage sludge with high ash content for biochar production and Cu (II) sorption. Sci. Total Environ..

[39] Xu C.-Y., Li Q.-R., Geng Z.-C., Hu F.-N., Zhao S.-W. (2020). Surface properties and suspension stability of low-temperature pyrolyzed biochar nanoparticles: Effects of solution chemistry and feedstock sources. Chemosphere.

[40] Luo S., Qin J., Wu Y., Feng F. (2022). Tetracycline adsorption on magnetic sludge biochar: Size effect of the Fe_3_O_4_ nanoparticles. R. Soc. Open Sci..

[41] Wang Z., Shen D., Shen F., Li T. (2016). Phosphate adsorption on lanthanum loaded biochar. Chemosphere.

[42] Useviciute L., Baltrenaite E. (2020). Methods for Determining Lignocellulosic Biochar Wettability. Waste Biomass Valorization.

[43] Moradi-Choghamarani F., Moosavi A.A., Sepaskhah A.R., Baghernejad M. (2019). Physico-hydraulic properties of sugarcane bagasse-derived biochar: The role of pyrolysis temperature. Cellulose.

[44] Jayalakshmi A., Rajesh S., Senthilkumar S., Mohan D. (2012). Epoxy functionalized poly(ether-sulfone) incorporated cellulose acetate ultrafiltration membrane for the removal of chromium ions. Sep. Purif. Technol..

[45] Lundy R., Byrne C., Bogan J., Nolan K., Collins M.N., Dalton E., Enright R. (2017). Exploring the Role of Adsorption and Surface State on the Hydrophobicity of Rare Earth Oxides. Acs Appl. Mater. Interfaces.

[46] Seo J., Moon J., Kim J.H., Lee K., Hwang J., Yoon H., Yi D.K., Paik U. (2016). Role of the oxidation state of cerium on the ceria surfaces for silicate adsorption. Appl. Surf. Sci..

[47] Tan G., Liu Y., Xiao D. (2019). Preparation of manganese oxides coated porous carbon and its application for lead ion removal. Carbohydr. Polym..

[48] Wang C.Q., Wang H. (2018). Pb(II) sorption from aqueous solution by novel biochar loaded with nano-particles. Chemosphere.

[49] Song J.-M., Chen S.-Y., Shen Y.-L., Tsai C.-H., Feng S.-W., Tung H.-T., Chen I.-G. (2013). Effect of surface physics of metal oxides on the ability to form metallic nanowires. Appl. Surf. Sci..

[50] Kumar S., Ahmed F., Ahmad N., Shaalan N.M., Kumar R., Alshoaibi A., Arshi N., Dalela S., Alvi P.A., Kumari K. (2022). Influence of Fe and Cu Co-Doping on Structural, Magnetic and Electrochemical Properties of CeO_2_ Nanoparticles. Materials.

[51] Jia Y., Zhang Y., Fu J., Yuan L., Li Z., Liu C., Zhao D., Wang X. (2019). A novel magnetic biochar/MgFe-layered double hydroxides composite removing Pb^2+^ from aqueous solution: Isotherms, kinetics and thermodynamics. Colloids Surf. A-Physicochem. Eng. Asp..

[52] Yang T., Xu Y., Huang Q., Sun Y., Liang X., Wang L. (2022). Removal mechanisms of Cd from water and soil using Fe-Mn oxides modified biochar. Environ. Res..

[53] Liang T., Li L., Zhu C., Liu X., Li H., Su Q., Ye J., Geng B., Tian Y., Sardar M.F. (2020). Adsorption of As(V) by the Novel and Efficient Adsorbent Cerium-Manganese Modified Biochar. Water.

[54] Li M., Liu H., Chen T., Dong C., Sun Y. (2019). Synthesis of magnetic biochar composites for enhanced uranium (VI) adsorption. Sci. Total Environ..

[55] He S., Wu L., Zeng Y., Jia B., Liang L. (2022). Preparation of Ce-Ag bimetallic modified biochar composite for the efficient removal of sulfathiazole and its mechanism. Mater. Today Commun..

[56] Matharu J., Cabailh G., Lindsay R., Pang C.L., Grinter D.C., Skala T., Thornton G. (2011). Reduction of thin-film ceria on Pt(111) by supported Pd nanoparticles probed with resonant photoemission. Surf. Sci..

[57] Sun C., Chen T., Huang Q., Wang J., Lu S., Yan J. (2019). Enhanced adsorption for Pb(II) and Cd(II) of magnetic rice husk biochar by KMnO4 modification. Environ. Sci. Pollut. Res..

[58] Kypritidou Z., El-Bassi L., Jellali S., Kinigopoulou V., Tziritis E., Akrout H., Jeguirim M., Doulgeris C. (2022). Lead removal from aqueous solutions by olive mill wastes derived biochar: Batch experiments and geochemical modelling. J. Environ. Manag..

[59] Tiwari D., Laldanwngliana C., Choi C.-H., Lee S.M. (2011). Manganese-modified natural sand in the remediation of aquatic environment contaminated with heavy metal toxic ions. Chem. Eng. J..

[60] Aran D., Antelo J., Fiol S., Macias F. (2016). Influence of feedstock on the copper removal capacity of waste-derived biochars. Bioresour. Technol..

[61] Wu J., Wang T., Wang J., Zhang Y., Pan W.P. (2021). A novel modified method for the efficient removal of Pb and Cd from wastewater by biochar: Enhanced the ion exchange and precipitation capacity. Sci. Total Environ..

[62] Yao C., Zhu C. (2020). A new method of characterizing mass transfer controlling mechanism in pollutant adsorption from aqueous solutions. J. Mol. Liq..

[63] Zhang Z., Yu H., Zhu R., Zhan X., Yan L. (2020). Phosphate adsorption performance and mechanisms by nanoporous biochar-iron oxides from aqueous solutions. Environ. Sci. Pollut. Res..

[64] Li J., Li B., Huang H., Zhao N., Zhang M., Cao L. (2020). Investigation into lanthanum-coated biochar obtained from urban dewatered sewage sludge for enhanced phosphate adsorption. Sci. Total Environ..

[65] Gao L.-Y., Deng J.-H., Huang G.-F., Li K., Cai K.-Z., Liu Y., Huang F. (2019). Relative distribution of Cd2+ adsorption mechanisms on biochars derived from rice straw and sewage sludge. Bioresour. Technol..

[66] Liu P., Rao D., Zou L., Teng Y., Yu H. (2021). Capacity and potential mechanisms of Cd(II) adsorption from aqueous solution by blue algae-derived biochars. Sci. Total Environ..

[67] Sheng G., Wang S., Hu J., Lu Y., Li J., Dong Y., Wang X. (2009). Adsorption of Pb(II) on diatomite as affected via aqueous solution chemistry and temperature. Colloids Surf. A-Physicochem. Eng. Asp..

[68] Hou J., Ci H., Wang P., Wang C., Lv B., Miao L., You G. (2018). Nanoparticle tracking analysis versus dynamic light scattering: Case study on the effect of Ca^2+^ and alginate on the aggregation of cerium oxide nanoparticles. J. Hazard. Mater..

[69] El-Deen G.E.S., El-Deen S.E.A.S. (2016). Kinetic and isotherm studies for adsorption of Pb(II) from aqueous solution onto coconut shell activated carbon. Desalin. Water Treat..

[70] Li Q., Gao Y., Lang J., Ding W., Yong Y. (2017). Removal of Pb(II) and Cu(II) from aqueous solutions by ultraviolet irradiation-modified biochar. Desalin. Water Treat..

[71] Anirudhan T.S., Sreekumari S.S. (2011). Adsorptive removal of heavy metal ions from industrial effluents using activated carbon derived from waste coconut buttons. J. Environ. Sci..

[72] Phuong-Thao H., Ngoc-Tuan N., Ha Nguyen V., Phuong-Tung N., Trinh Duy N., Van-Phuc D. (2020). Modeling and optimization of biosorption of lead (II) ions from aqueous solution onto pine leaves (Pinus kesiya) using response surface methodology. Desalin. Water Treat..

[73] Shi S., Xu C., Wang X., Xie Y., Wang Y., Dong Q., Zhu L., Zhang G., Xu D. (2020). Electrospinning fabrication of flexible Fe_3_O_4_ fibers by sol-gel method with high saturation magnetization for heavy metal adsorption. Mater. Des..

[74] Zahedifar M., Seyedi N., Shafiei S., Basij M. (2021). Surface-modified magnetic biochar: Highly efficient adsorbents for removal of Pb(II) and Cd(II). Mater. Chem. Phys..

[75] Wang L., Wang J., Wang Z., He C., Lyu W., Yan W., Yang L. (2018). Enhanced antimonate (Sb(V)) removal from aqueous solution by La-doped magnetic biochars. Chem. Eng. J..

[76] Tan G., Wu Y., Liu Y., Xiao D. (2018). Removal of Pb(II) ions from aqueous solution by manganese oxide coated rice straw biochar—A low-cost and highly effective sorbent. J. Taiwan Inst. Chem. Eng..

[77] Ji Y., Zheng N., An Q., Sun S., Wang S., Li X., Li P., Hua X., Dong D., Zhao C. (2022). The effect of carbonization temperature on the capacity and mechanisms of Cd(II)-Pb(II) mix-ions adsorption by wood ear mushroom sticks derived biochar. Ecotoxicol. Environ. Saf..

[78] Yamada S. (2020). Cation-pi interactions in organic crystals. Coord. Chem. Rev..

[79] Zhu L., Zhao N., Tong L., Lv Y. (2018). Structural and adsorption characteristics of potassium carbonate activated biochar. Rsc Adv..

[80] Zhang C.-L., Qiao G.-L., Zhao F., Wang Y. (2011). Thermodynamic and kinetic parameters of ciprofloxacin adsorption onto modified coal fly ash from aqueous solution. J. Mol. Liq..

[81] Zhao D., Qiu S.K., Li M.M., Luo Y., Zhang L.S., Feng M.H., Yuan M.Y., Zhang K.Q., Wang F. (2022). Modified biochar improves the storage capacity and adsorption affinity of organic phosphorus in soil. Environ. Res..

